# Dynamic External Pelvimetry Test in Third Trimester Pregnant Women: Shifting Positions Affect Pelvic Biomechanics and Create More Room in Obstetric Diameters

**DOI:** 10.7759/cureus.13631

**Published:** 2021-03-01

**Authors:** Marco Siccardi, Cristina Valle, Fiorenza Di Matteo

**Affiliations:** 1 Obstetrics and Gynecology, Primal Osteopathy Institute, Savona, ITA; 2 Obstetrics and Gynecology, San Paolo Hospital, Savona, ITA; 3 Obstetrics and Gynaecology, San Paolo Hospital, Savona, ITA; 4 Yoga and Cranial Osteopathy, Primal Osteopathy Institute, Savona, ITA

**Keywords:** obstetrics, labor, pelvimetry, pregnancy, dystocia, childbirth, pelvis, biomechanics

## Abstract

Dystocia in labor is still a clinical challenge. The "contracted pelvis" is the absence of pelvic mobility, which leads to fetal-pelvic disproportion, obstructed labor, and operative delivery. Maternal pelvis biomechanics studies by high technological techniques have shown that maternal shifting positions during pregnancy and labor can create more room in the pelvis for safe delivery. The external and internal pelvic diameters are related. The present study aims to evaluate the external obstetric pelvic diameters in shifting positions using a clinical technique suitable for daily practice in every clinical setting: the dynamic external pelvimetry test (DEP test). Seventy pregnant women were recruited, and the obstetric external pelvic diameters were measured, moving the position from kneeling standing to "hands-and-knees" to kneeling squat position. Results showed modification of the pelvic diameters in shifting position: the transverse and longitudinal diameters of Michaelis sacral area, the inter-tuberosities diameter, the bi-trochanters diameter, and the external conjugate widened; the bi-crestal iliac diameter, the bi-spinous iliac diameter, and the base of the Trillat's triangle decreased. The test showed good reproducibility and reliability. Linear correlations were found between diameters and between the range of motion of the diameters. The maternal pelvis is confirmed to modify the diameters changing its tridimensional shape. The pelvic inlet edge's inclination is inferred to be modified, facilitating the fetal descend. The pelvic outlet enlarged the transverse diameter, facilitating birth. The DEP test estimates the pelvic diameters' modification with postural changes, as magnetic resonance (MR) and computational biomechanics studies have demonstrated.

## Introduction

Childbirth is still a potentially complicated event for the woman and the newborn, with destructive health consequences in low-resource countries [1]. It is mainly due to cephalo-pelvic disproportion (CPD): absolute fetal-pelvic disproportion is uncommon, but the cesarean section for CPD indication is typical [1]. The "contracted pelvis" is the absence of pelvic mobility, which leads to fetal-pelvic disproportion, obstructed labor, dystocia, and operative delivery. The pelvic biomechanics and physiology during pregnancy, labor, and delivery have not yet been fully understood. Studies and research with magnetic resonance (MR), optoelectronic devices, and 3-D reconstruction computational analysis attempted to elucidate the biomechanics and dynamics of the maternal pelvic tissue anatomy during pregnancy for safe childbirth [2-8]. The freedom to move and standing positions throughout labor allows women to have better comfort during labor and the labor to proceed physiologically [9,10]. Shifting positions enables women to modulate the pelvic inlet, midlet, and outlet space, influencing and assisting the fetal head's passage during the descent. Permitting more area in the birth canal's diameters will support the fetal presenting part's engagement. In the upright birthing positions, the maternal pelvis will offer less resistance to the force of progression in the pathway towards birth.

Obstetric pelvimetry has been used to evaluate the birth canal's childbirth capacity's endopelvic spaces in static conditions. Clinical studies have correlated the external pelvic diameter's raw static dimension with the internal diameters and the risk of "contracted pelvis" and obstructed labor [7,8,11-13]. In more recent years, the maternal pelvis's biomechanics begun to be studied in different postures to elucidate the birth canal's anatomical dynamic physiology. Studies have aimed to evaluate the change of the internal pelvic spaces during the maternal shifting positions. Research performed with MR and optoelectronic devices in pregnant and non-pregnant women showed an increase in the pelvis internal and external diameters and a variation in the lumbosacral angle with the hip joints' degree flexion [2-6]. High technologies are not available in daily practice. The Dynamic External Pelvimetry test (DEP test) was defined to evaluate the external obstetrics pelvic diameters in shifting positions. It is a manual examination, using a simple instrument, available for obstetrics routine practice in every type of clinical setting [14]. Our previous preliminary studies have shown a variation of the Michaelis sacral rhombus's and the external pelvimetry's diameters with the maternal posture change [14-16].

The present study in a group of pregnant women in their third trimester of pregnancy is part of more extensive research on osteopathy for pregnancy. It observes the external diameters of obstetric pelvimetry assessed in shifting positions using the DEP test and considers the biomechanical implications for the birth canal's pelvis mobility. The present paper is addressed to evaluate and analyze the modification of the external pelvic diameters' measurement with maternal position change and confront literature data results. Moreover, the study considers the accuracy of measurements from the intra-observer and interobserver agreement, the intra-diameter and inter-diameter measurements' linear correlation in different positions, and the intra-diameter and inter-diameter range of movement (ROM) relationship while shifting positions.

## Materials and methods

The cross-sectional study was performed at the Obstetrics Department of San Paolo Hospital of Savona, Italy (EU) and followed the Strengthening the Reporting of Observational Studies in Epidemiology (STROBE) statement checklist. Seventy low-risk pregnant women in their third trimester were recruited at the Clinic for Physiological Pregnancy of the Obstetrics Department between 10 January and 30 October 2020. They gave their informed consent before the study; the low-risk pregnancy condition was certified from the medical records. Only the subjects who did not report abdominal, pelvic, or vertebral surgical interventions, did not suffer from lumbopelvic pains, did not have previous obstetrics complications, and did not have abdominal-pelvic diseases at the clinical evaluation were accepted. The authors contributed to define the protocol from the previous study [14]. Data were stored before the statistical analysis in a raw data deposit file (doi: 10.13140/RG.2.2.33800.03847). The study was conducted in conformity with the Helsinki Declaration. It was part of the Osteopaths in Obstetrics study and updating program (the Institutional Board of San Paolo Hospital issued approval n. 119933/2019). The number of subjects recruited exceeds what is suggested in biomechanical studies concerning the significant effect of the 0.80 statistical power [6,17].

**Figure 1:**
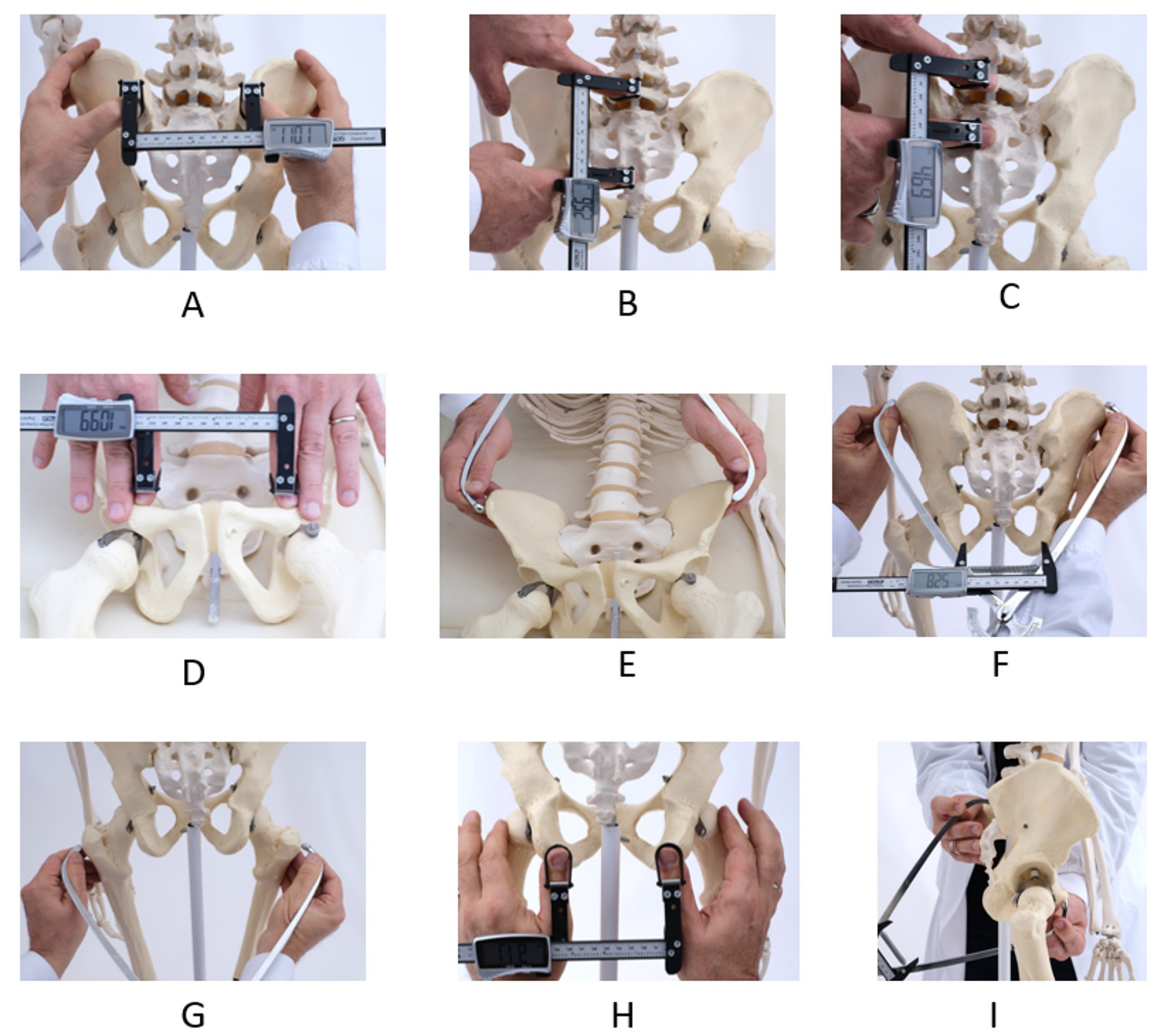
The diameters of the Dynamic External Pelvimetry test. A: the transverse diameter of the sacral area. B: the longitudinal diameter of the sacral area. C: the longitudinal hemi-diameter of the sacral area. D: the base of Trillat's triangle. E: the iliac bi-spinous diameter. F: the iliac bi-crestal diameter. G: the bi-trochanters diameter. H: the ischial inter-tuberosities diameter. I: the external obstetric conjugate.

The authors registered the patients' general characteristics and measured the external pelvic diameters in the three different postural positions using the DEP test [14]. The first author evaluated the posterior and anterior pelvic diameters of the external obstetric pelvimetry. The posterior pelvic diameters were: the transverse diameter of the sacral area of Michaelis (between the posterior superior iliac spines), the longitudinal diameter (between the tip of the spinous process of the fifth lumbar vertebra and the superior edge of the gluteal cleft, corresponding to the fourth-fifth sacral segment), and the longitudinal hemi-diameter (between the spinous process of the fifth lumbar vertebra and the second sacral segment) of the sacral rhombus of Michaelis, the inter-tuberosities diameter (between the ischial tuberosities), the external obstetric conjugate (the Baudelocque's diameter: between the first sacral segment and the upper border of the pubic symphysis), and the bi-trochanteric diameter (between the two femoral trochanters). The anterior pelvic diameters were the bi-crestal diameter (between the iliac crests), the base of the Trillat's triangle (on the upper edge of the pubis, between the inguinal ligaments' fold), and the iliac bi-spines diameter (between the outer edges of the anterior superior iliac spines) (Figure 1A-1I) [14].

**Figure 2:**
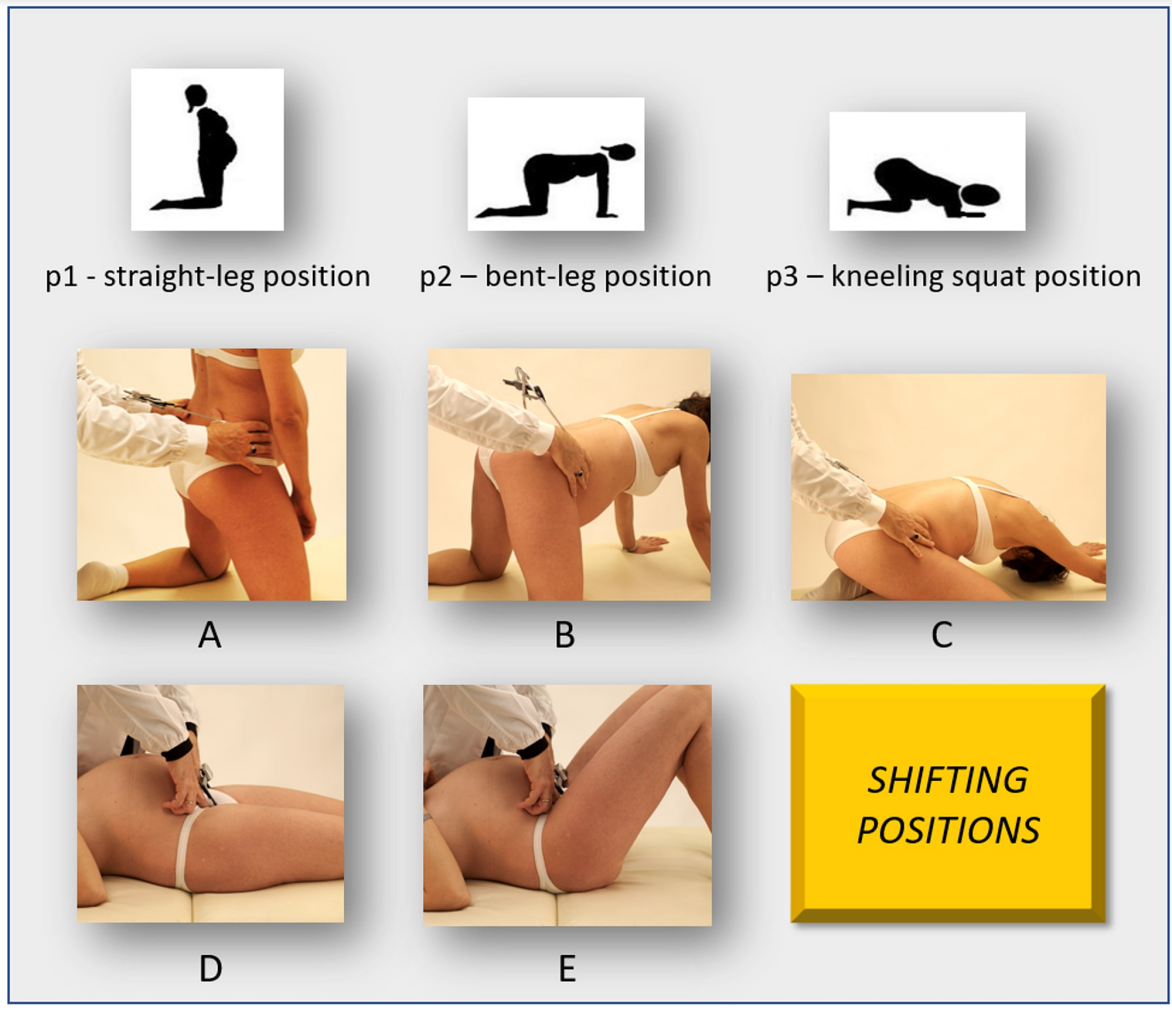
DEP test: shifting positions procedure. A, D: p1, straight-leg positions. A: standing kneeling position. D: supine position. B, E: p2, bent-leg positions. B: "all-fours" position. E: bent-leg supine position. C: p3, kneeling squat position.

The diameters were evaluated in shifting positions depending on the degree of hip flexion on the pelvis: p1 "kneeling erect" position (the erect straight-led position), p2 "hands-and-knees" position (the bent-leg position, with 90° flexed hips, or "all fours" position), and p3 "kneeling squat" position (Figures 2A, 2B, 2C). The measurement of the anterior diameters (the base of the Trillat's triangle and the bi-spinous diameter) has used the straight-leg supine position (p1), and the situation with the lower limbs flexed to the maximum, keeping the feet in contact with the table as close as possible at the buttocks (p2) (Figures 2D, 2E). The measurement device used was the CE patented instrument "BMK" (Metrica, Milan, Italy, EU) and the prototype accessory pelvimeter [14].

The study appraised the DEP test procedure reliability and reproducibility to steadily and firmly maintain the fingers’ contact on the diameter references during the patients’ movement. The ability to recognize the bone landmark was not a study's aim, but the operators' accordance in estimating pelvic diameters modification in shifting positions. The authors assessed the inter-observer (C.V.** **and F.D.M.) and intra-observer (M.S.) agreement from randomly selected diameters measured by direct finger contact on the bone landmark, starting from a known value. Each patient's diameter was randomly selected and measured in the different positions before and after each session to avoid bias.

The OpenOffice spreadsheet version 4.1.2 (Apache Software Foundation, Wakefield, MA, USA) was used to register the handwriting recorded measurements' values. Data of the pelvic diameters are expressed in millimeters and reported as mean (M), standard deviation (SD), minimum and maximum value, standard error of the mean (SEM), and 95% confidence interval (95% CI). The Kolmogorov-Smirnov test evaluated the normality of the data distribution. The t-test for repeated measurements (paired t-test), the intraclass correlation coefficient (ICC) and the Bland-Altman analysis, and the intra-diameter and inter-diameter Pearson's correlation coefficient with Fisher's Z were performed with the open-source statistical software StatsToDo (Statstodo Trading Pty Ltd, Brisbane, Queensland, Australia). A p-value <0.05 and an ICC >0.70 were considered significant.

## Results

The patients' general characteristics are reported and described as mean and standard deviation (Table 1). 100% diameters had permitted to be measured. 1,509 out of 1,610 measurements were recorded for the statistical analysis: 101 measures were eliminated for procedural errors or difficult handwriting interpretation. The statistical analysis of the external pelvic diameters measured began with the Kolmogorov-Smirnov test, highlighting the normality of each diameter data distribution, with a correlation between expected and actual cumulative frequency >0.98.

**Table 1:** General characteristics of the pregnant group Data are shown as mean and standard deviation (SD) or absolute value and rate (%). Estimated fetal weight from Johnson's formula. Complicated deliveries were those who needed medical assistance, not safe or available in a home-birth setting, or limited resource clinic (prostaglandins labor induction, first-stage oxytocin augmentation or induction, epidural analgesia, operative delivery, cesarean section).

	Nulliparas (n 52)	Multiparas (n 18)
Weeks of gestation	34.6 (2.9)	34,7 (2.5)
Age (years)	31.6 (4.3)	36,3 (3.2)
Height (cm)	164.5 (5.9)	162,2 (6.9)
Actual weight (kg)	68.2 (9.2)	70,1 (13.4)
Pre-pregnancy weight (kg)	58.1 (8.7)	62,2 (14.7)
Weight gain (kg)	10.1 (3.5)	9,2 (5.1)
Uterine symphysis-fundus length (cm)	33.9 (4.1)	34,2 (4.1)
Estimated fetal weight (gm)	3106.1 (1063.7)	3203.3 (599.7)
Natural deliveries (n)	42 (80.7%)	15 (83.3%)
Complicated deliveries (n)	10 (19.3%)	3 (16.7%)

**Figure 3:**
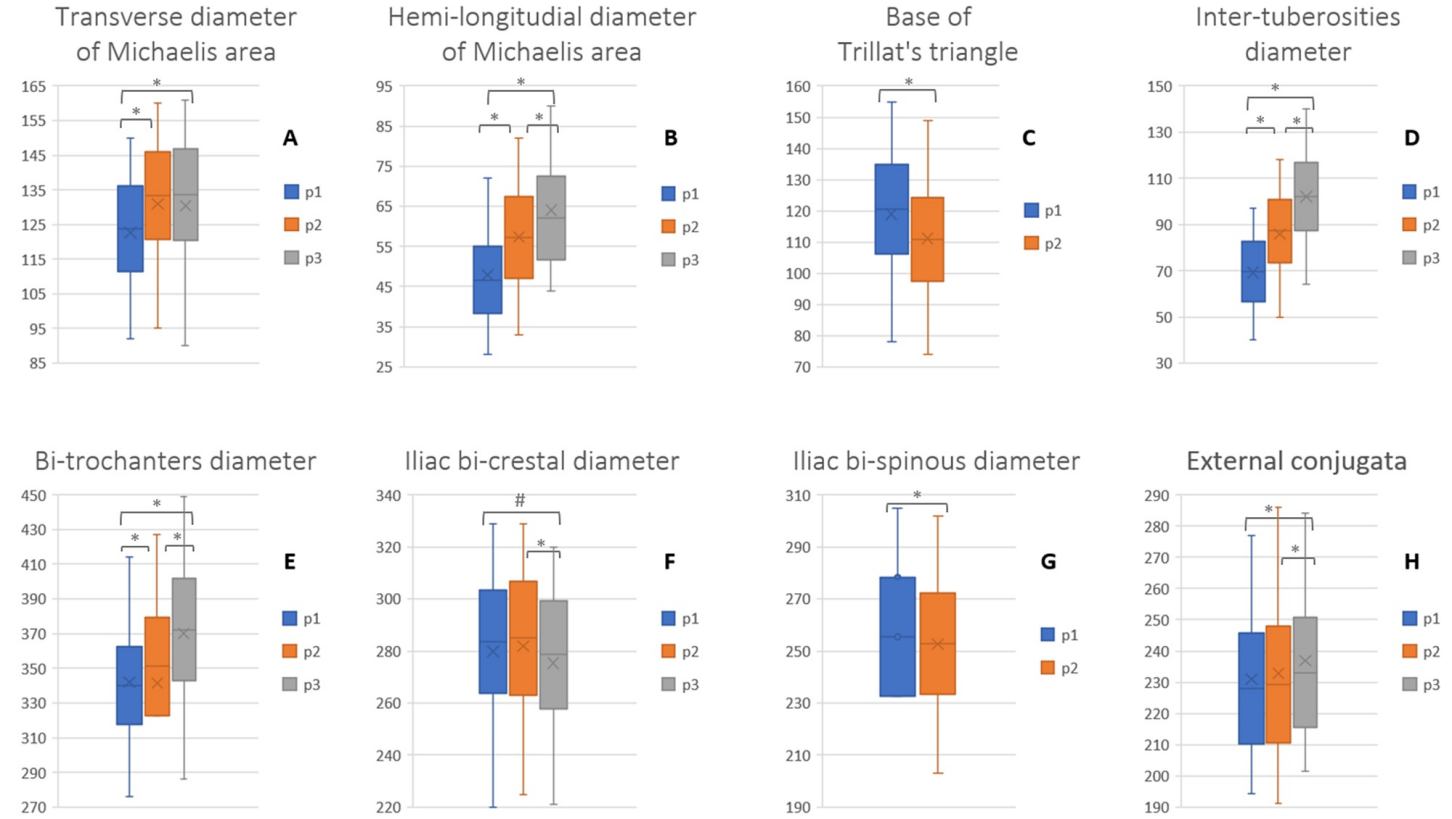
Graphic representation of the values of the pelvic diameter in shifting positions Data are shown in millimeters as mean and standard deviation, minimum and maximum. The paired t-test was statistically significant for all the diameters, not in all positions. p1: straight-leg position. p2: bent-leg position. p3: squat position. *p-value <0.0001 #p-value = 0.0006

The external pelvic diameters showed a gradual difference in dimension from the straight-leg position (p1) to the 90° bent-leg (p2) and the kneeling squat position (p3) (Table 2). The transverse diameter of the Michaelis sacral rhomboid area increased between the straight-leg and bent-leg position (p2-p1: mean of difference 9.5 mm; SD 4.3; SEM 0.5; 95% CI 8.5-10.5 ; p <0.0001); between the "all fours" and squat position the range was not statistically significant (Figure 3A). Multiparous patients had a larger ROM from p1 to p2 position than nulliparous. The Michaelis area's longitudinal hemi-diameter continuously increased its length from p1 to p2 to p3 (Figure 3B). It showed a statistically significant difference between nulliparous and multiparous, with greater diameters in the multiparas and a wider ROM between the p1 and p2 positions.

**Table 2:** The biodynamics of the obstetrics external pelvic diameters in shifting positions Data are reported in millimeters as mean and standard deviation (SD), minimum and maximum value.

Diameter (mm)	p1-straight legs		p2-90° bent legs		p3-kneeling squat		Paired t-test
	mean (SD)	min-max	mean (SD)	min-max	mean (SD)	min-max	p1 vs. p3
Transverse Michaelis diameter	123.8 (12.3)	92-150	133.3 (12.6)	95-160	133.6 (13.1)	90-160	p<0.0001
Hemi-longitudinal Michaelis diameter	46.6 (8.3)	28-72	57.2 (10.1)	33-82	62.1 (10.4)	44-90	p<0.0001
Inter-tuberosities diameter	69.6 (12.9)	40-97	87.2 (13.6)	50-118	102.1 (14.7)	64-140	p<0.0001
Bi-trochanteric diameter	340.1 (22.2)	228-414	351.1 (28.4)	307-427	372.2 (29.4)	316-449	p<0.0001
Iliac bi-crestal diameter	283.4 (19.8)	220-329	285.1 (21.8)	225-329	278.5 (20.6)	221-320	p= 0.0006
External conjugate	226.3 (21.4)	133-277	227.6 (22.3)	132-286	231.5 (20.8)	141-284	p<0.0001
Iliac bi-spinous diameter	257.2 (18.5)	146-305	252.5 (19.4)	143-302			p<0.0001
The base of Trillat’s triangle	120.6 (14.3)	78-154	110.9 (13.3)	74-149			p<0.0001
Longitudinal Michaelis diameter	108.8 (16.4)	62-156					

The range of motion of the base of the Trillat's triangle was measured in the supine position. The difference in measurements decreases between the p1 and p2 positions, reporting a mean difference of 9.7 mm (SD 4.7; SEM 0.5; 95% CI 8.5-10.8; p <0.0001) (Figure 3C). The inter-tuberosities and bi-trochanters diameter increased the measures passing from position p1 to p2 and p2 to p3 (Figure 3D, 3E). The iliac crest distance showed a small increase between the standing position and the "all fours", which was not statistically significant but decreased significantly to the kneeling squat position (Figure 3F). The external conjugate reported a not statistically significant increase between p1 and p2, but it enlarges significantly to the kneeling squat position (Figure 3H). The Baudelocque and the bi-crestal diameter showed greater but not statistically significant values and broader increases in the multiparous than nulliparous women in all the positions. The distance between the two superior anterior iliac spines evaluated in the supine position changed significantly from p1 to p2 (Figure 3G).

**Figure 4:**
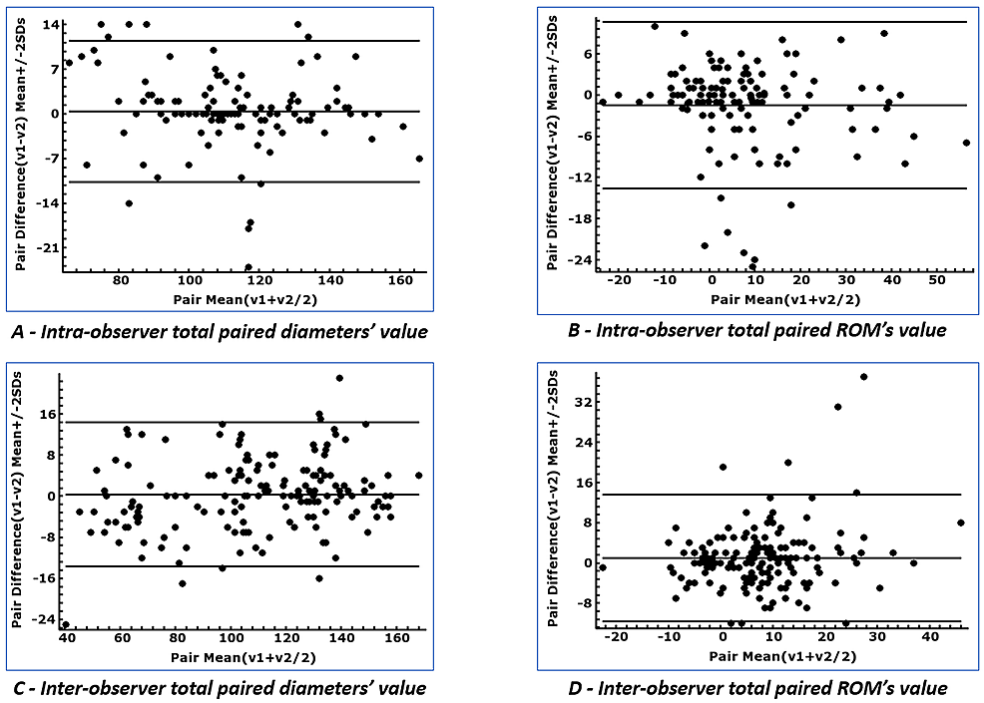
Intra-observer and inter-observer agreement A, B: Intra-observer Bland-Altman plot showing mean difference ±2 standard deviation (SD) values (v). C, D: inter-observer Brand-Altman plot showing mean difference ±2 SD values (v). ROM: the range of motion.

The Bland-Altman plot and analysis evaluated the DEP test measurement technique reproducibility and reliability from 648 paired pelvic diameters and ROMs' values (Figure 4). The intra-observer ICC showed perfect agreement in diameters' measurement and diameters' range of movement (Table 3). The inter-observer diameters' measurement reliability showed an excellent coefficient, and the ROMs demonstrated good concordance proven by ICC >0.75 (Table 4).

**Table 3:** Intra-observer agreement table. ICC: intraclass correlation coefficient. SEM: standard error of the mean. 95% CI: 95% confidence interval. ROM: range of motion

	ICC	Bland-Altman analysis		
		Mean pair difference (mm)	SEM	95% CI
p1 diameters	0.98	1.6275	0.6831	0.2554 to 2.9996
p2 diameters	0.98	0.6863	0.6713	-0.662 to 2.0346
p3 diameters	0.97	-1.2889	0.9856	-3.2752 to 0.6974
Total paired diameters (n= 155)	0.96	0.4082	0.4564	-0.4939 to 1.3103
p1-p2 ROM	0.94	-0.9412	0.6936	-2.3344 to 0.452
p1-p3 ROM	0.90	-2.2444	1.0849	-4.4308 to -0.058
p2-p3 ROM	0.84	-1.6	0.9526	-3.5199 to 0.3199
Total paired ROMs’ values (n= 148)	0.91	-1.5674	0.5228	-2.601 to -0.5338

**Table 4:** Inter-observer agreement table ICC: intraclass correlation coefficient. SEM: standard error of the mean. ROM: range of motion. 95%CI: 95% confidence interval.

	ICC	Bland-Altman analysis		
		Mean pair difference (mm)	SEM	95% CI
p1 diameters	0.97	-0.1864	0.8473	-1.8825 to 1.5097
p2 diameters	0.97	0.322	0.8755	-1.4305 to 2.0745
p3 diameters	0.95	1	1.0577	-1.1198 to 3.1198
Total paired diameters (n= 174)	0.97	0.3678	0.5335	-0.6851 to 1.4207
p1-p2 ROM	0.75	0.4746	0.762	-1.0506 to 1.9998
p1-p3 ROM	0.77	1.4464	1.093	-0.7442 to 3.637
p2-p3 ROM	0.85	1.0179	0.5869	-0.1583 to 2.1941
Total paired ROM’s values (n= 171)	0.81	0.9708	0.482	0.0192 to 1.9224

Pearson's correlation coefficient (with Fisher's Z) analyzed the absolute values between the pelvic diameters (inter-diameter correlation) in each different position (Table 5). It generally has shown direct relationships between diameters in changing positions. The inter-diameter ROM correlation was analyzed (Table 6), showing a close relationship between the external conjugate's ROM and the transverse diameter of Michaelis rhombus's ROM. The intra-diameter analysis indicated the strong interrelation between the different values of a single diameter in each position examined. The discrete values of a diameter measured in a position showed full accordance with the measurements obtained in other positions (p1 and p2, p1 and p3, p2 and p3, with r>0.80 and p<0.0001). The intra-diameter ROM relationship, that is, the linear correlation between the changes in measurement between one position and the next, was calculated. It permits a clinical setting to evaluate diameters' ROM more rapidly and comfortably for patients (Table 7). 

**Table 5:** inter-diameter correlation in shifting position Statistically significant Pearson's Correlation Coefficient between diameters.

Diameter	p1 - Straight-leg position	p2 - Bent-leg position	p3 - Kneeling squat position
Transverse Michaelis area	Longit. Michaelis, Iliac bi-crestal, Bi-trochanteric	Inter-tuberosities, Bi-crestal, Bi-trochanteric, Iliac bi-spinous	Inter-tuberosities, Iliac bi-crestal
Hemi-longit. Michaelis area	Inter-tuberosities, Iliac bi-crestal, Base Trillat’s triangle	Inter-tuberosities, Iliac bi-crestal	Inter-tuberosities, Bi-trochanteric
Inter-tuberosities	Base Trillat’s triangle, Hemi-longit. Michaelis	Base Trillat’s triangle Hemi-longit. Michaelis Transverse Michaelis	Hemi-longit. Michaelis, Transverse Michaelis
External conjugate	Iliac bi-crestal, Bi-trochanteric	Iliac bi-crestal, Bi-trochanteric	Iliac bi-crestal, Bi-trochanteric
Iliac bi-crestal	Bi-trochanteric, Iliac bi-spinous, Base Trillat’s triangle, Hemi-longit. Michaelis, Transv Michaelis, External Conjugate	Bi-trochanteric, Iliac bi-spinous, Hemi-longit Michaelis, Transv Michaelis, External Conjugate	Bi-trochanteric, Transv Michaelis, External Conjugate
Bi-trochanters	Iliac bi-crestal, Iliac bi-spinous, Hemi-longit. Michaelis, Transv Michaelis, External Conjugate	Iliac bi-crestal, Iliac bi-spinous Base Trillat’s triangle, Transv Michaelis, External Conjugate	Iliac bi-crestal, Hemi-longit. Michaelis, External Conjugate
Iliac bi-spinous	Iliac bi-crestal, Bi-trochanters, Base Trillat’s triangle	Iliac bi-crestal, Bi-trochanters, Base Trillat’s triangle, Transv Michaelis	
Base Trillat’s triangle	Iliac bi-crestal, Iliac bi-spinous, Hemi-longit Michaelis, Inter-tuberosities	Iliac bi-crestal, Iliac bi-spinous, Inter-tuberosities	

**Table 6:** Inter-diameter ROM's correlation in shifting position. Statistically significant Pearson's Correlation Coefficient between ROM's diameters. ROM: range of motion.

Diameter	p1-p2 ROM	p1-p3 ROM	p2-p3 ROM
Transverse Michaelis area	Hemi-longit. Michaelis, External conjugate	Iliac bi-crestal, Bi-trochanteric, External conjugate	External conjugate, Iliac bi-crestal
Hemi-longit. Michaelis area	Transverse Michaelis		Inter-tuberosities, Bi-trochanteric
Inter-tuberosities			Hemi-longit. Michaelis
External conjugate	Base Trillat’s triangle, Transverse Michaelis	Transverse Michaelis	Transverse Michaelis
Iliac bi-crestal		Bi-trochanteric, Transverse Michaelis	Transverse Michaelis
Bi-trochanters		Iliac bi-crestal, Transverse Michaelis	Hemi-longit. Michaelis
Base Trillat’s triangle	External conjugate		

**Table 7:** Intra-diameter ROM's Pearson's Correlation Coefficient. N.S.: not statistically significant. ROM: range of motion.

Diameter	Correlation test between p1-p2 ROM and p2-p3 ROM	Correlation test between p1-p2 ROM and p1-p3 ROM
Transverse Michaelis	N.S.	r = 0.7896 with 95%CI 0.681-0.8643; Z = 1.0705 with 95%CI 0.831-1.3099 (p <0.0001)
Hemi-longitudinal Michaelis	N.S.	r = 0.7121 with 95%CI 0.5666-0.8145; Z = 0.8914 with 95%CI 0.6424-1.1403 (p <0.0001)
Inter-tuberosities	r = 0.2598 with 95%CI 0.017-0.4737; Z = 0.2659 with 95%CI 0.017 -0.5149 (p = 0.03)	r = 0.794 with 95%CI 0.6822-0.8695; Z = 1.0821 with 95%CI 0.8331-1.331 (p <0.0001)
External conjugate	N.S.	r = 0.7004 with 95%CI 0.5492-0.8073; Z = 0.8681 with 95%CI 0.6172-1.1191 (p <0.0001)
Iliac bi-crestal	r = -0.445 95%CI -0.6227 to -0.2237; Z = -0.4785 with 95%CI -0.7294 to -0.2275 (p = 0.0002)	r = 0.7318 with 95%CI 0.5926-0.8286; Z = 0.9326 with 95%CI 0.6817-1.1836 (p <0.0001)
Bi-trochanters	N.S.	r = 0.5497 with 95%CI 0.3458-0.7041; Z = 0.618 with 95%CI 0.3606-0.8753 (p <0.0001)

## Discussion

The external pelvic diameters evaluated through the DEP test on 70 pregnant women in their third trimester showed a statistically significant adaptation in the different positions studied, homogeneous with literature data [2-6]. The test procedure has significant repeatability and reliability, fundamental in the clinical setting for screening women at risk pelvic hypomobility. Linear relationships are found between diameters' measures and range of motion in shifting positions, denoting functional symmetric pelvic balance in loading situations. The DEP test instruments are simple tools potentially available in every clinical setting. External pelvimetry is part of the cultural background of obstetric health care operators, and the DEP test is a non-invasive postural test convenient to perform [15].

External obstetric pelvimetry was founded for the early diagnosis of mechanical dystocia. It is a clinical procedure for daily practice to detect the narrow pelvis and consequent fetal-pelvic disproportion implicated in labor arrest childbirth complications. Pelvimetry has been complemented and replaced by radiological pelvimetry, which closer evaluated the birth canal's diameters. Researches observed correspondence between the external and internal pelvic diameters (Table 8) [12,14,18,19]. Computed tomography (CT) and MR displaced X-ray pelvimetry but did not enter in daily practice like ultrasound: the static pelvimetry evaluation has not resulted predictive for dystocia. Childbirth and delivery are dynamic processes. Pelvic mobility is one of the labor factors and permits safe childbirth: the contracted pelvis leads to obstructed labor and operative deliveries [2-6,9,10]. The freedom to move, the standing positions throughout labor, and giving birth in non-recumbent situations allow women to have better comfort during labor and labor to proceed physiologically [9,10]. Studies demonstrated the pelvic space diameters adaptation to patients' postures [2-6].

**Table 8:** Relationship between external and internal pelvic diameters from literature data.

External Pelvic Diameters	Internal Pelvic Diameters
External conjugate	Obstetric conjugate
Inter-tuberosities diameter, Bi-trochanteric diameter	Transverse pelvic outlet diameter
The base of Trillat’s triangle	Interspinous diameter, transverse pelvic midlet
Anterior superior iliac spine diameter	Transverse pelvic inlet diameter
Posterior superior iliac spine diameter, the transverse diameter of the Michaelis sacral area	Transverse pelvic inlet diameter
Modified Schober test, the hemi-longitudinal diameter of Michaelis area	Lumbosacral flexion degree (promontory angle)

Biomechanical research has highlighted an appreciable movement of the sacroiliac joints, the lumbosacral junction, and the pubic symphysis, which justifies the pelvis's ability to modify three-dimensionally its diameters and internal spaces [19-21]. It can be assumed that assessment of adequate pelvic mobility is pivotal in supporting vaginal deliveries and preventing labor dystocia. Few papers have investigated the mobility of pelvic diameters with position change, and the present study is the first regarding external pelvimetry evaluation in shifting positions. MR and optoelectronic studies have shown that the internal pelvic diameters and the sacral promontory angle change from the supine position to the "hands-and-knees" and the squat position [2-6]. Our study generally agrees with the results obtained from the complex high-technological methodologies (MR, optoelectronic trackers, and three-dimensional computerized reconstructions), which are not suitable for daily clinical practice.

The MR study of Michel et al. on 35 non-pregnant women resulted in a slight modification of the pelvic diameters in shifting positions [2]. The obstetric conjugate remained unchanged between the starting position and the "all-4" position. It decreased significantly in the squat position, the pelvic midlet and outlet diameters increased, and the transverse inlet diameter decreased. Reitter et al. demonstrated changes in 50 pregnant and 50 non-pregnant patients' internal pelvimetry consistent with Michel and colleagues' study [3]. MR images revealed a decreased obstetric and anatomical conjugate and increased transverse pelvic diameters and subpubic angle from the supine dorsal to the kneeling squat position. The authors reported difficulty measuring transverse diameters, which reached 44% failure for the pelvic outlet inter-tuberosities diameter due to the complexity in recognizing the right plane of reference for correct measurement [3].

The 3D computational reconstruction simulation research by Hemmerich et al. has reported a modification of pelvic diameters measured from the standing position to the squat position confronting Reitter and Michel's results [4,5]. The study disclosed that the anteroposterior diameters increased, the transversal inlet decreased, the transversal midlet and outlet increased, the superior pubic width decreased, and the inferior pubic angle increased. They stated that the most significant pelvic space was achieved during shifting positions. Consistent with their statement, our study shows that all the posterior pelvic diameters increase in the "hands-and-knees" position. Hemmerich and colleagues' simulating study had considered a woman's loading conditions on feet squat position without hands support, which is an unusual position for parturients to maintain for a long time [10]. They have emphasized the importance of the squat position on the feet to investigate pelvic biomechanics during the second stage of labor to prevent childbirth complications, such as obstructed labor [5]. The pelvic outlet is demonstrated to widen in the squat position, permitting space for the fetal head during the expulsive labor stage; our study agrees with instrumental research showing an evident increase of the transverse pelvic outlet diameter (inter-tuberosities and bi-trochanters diameter) [2-5].

Most obstructed labor and cephalo-pelvic disproportion are diagnosed during uterine cervix dilation in the first and active phase of labor, whilst the fetal head glides on the pelvic inlet for engaging in the pelvic midlet. Women often utilize the standing and kneeling "all-4" position during the first stage to release the uterine contraction pain and facilitate the fetal descend, but the squat positions are not recommended [10]. The anteroposterior and transverse inlet diameters need to modify to support the fetal descend favorably. The trunk's weight burdens the sacrum less in the intermediate "all-4" than the standing and squat position: peak increases in the simulated pelvic diameter measurement reconstruction were demonstrated during the movement's dynamic portion from standing to squat. [5]. The relaxin-induced joint ligaments' laxity could let the sacrum have more posterior mobility (contra-nutation movement) from the postural intra-abdominal hydrostatic pressure. The postural stress in shifting positions, mimicking the fetal head passing through the pelvic inlet, could maintain the sacral base relatively posterior and the Michaelis area transverse diameter wide [16].

**Figure 5:**
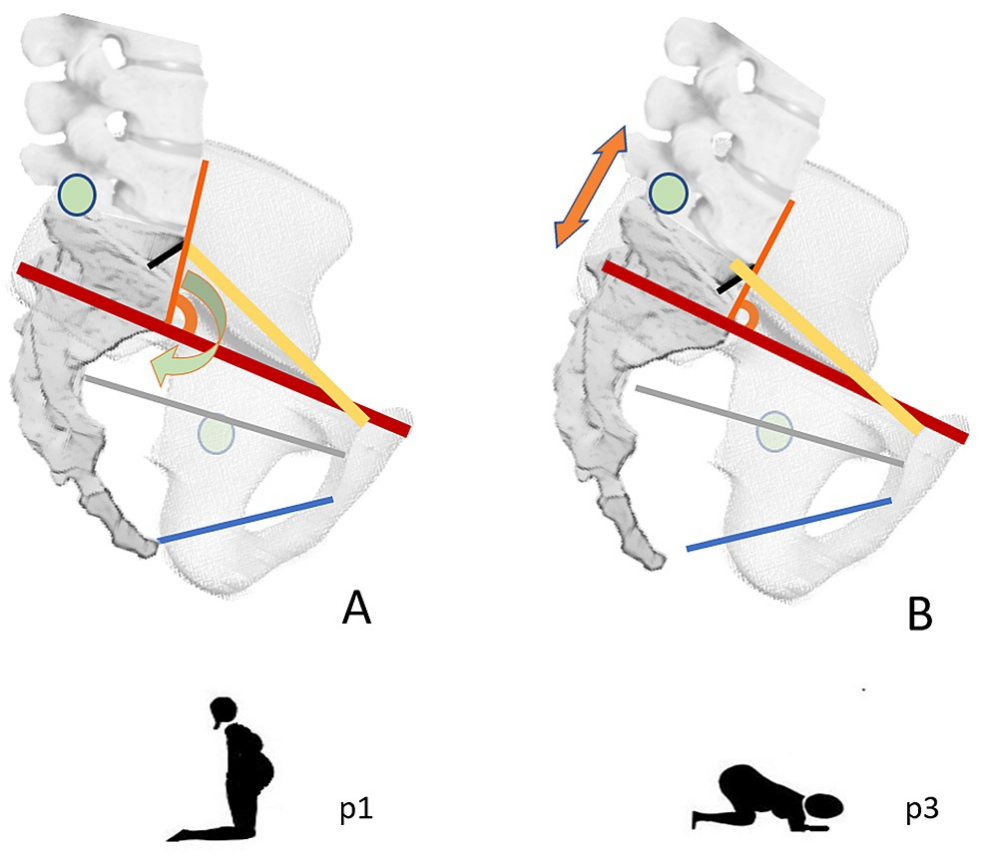
Pelvic anteroposterior diameters in shifting positions. A, p1: straight-leg position. B, p3: squat position. Red line: external conjugate measured in straight-leg position. Yellow line: obstetric conjugate measured in the straight-leg position. Grey line: midlet anteroposterior diameter measured in the straight-leg position. Blue line: outlet anteroposterior diameter measured in straight-leg position. Black line: anterior sacral promontory inclination, angle respect the lumbar spine anterior line. Orange line: lumbar spine anterior line. Orange arrows: the longitudinal hemi-diameter of the Michaelis area. Orange angle: the angle between the lumbar spine and the external conjugate, according to Desseauve et al. [6]. Green dots: pelvic movement fulcra (lumbar-sacral posterior zygoapophyseal joint and hip joint). Green curved arrow: the direction of the sacral base, according to Hemmerich et al. [5]. Graphic images of the pelvis sagittal sections show the biodynamics of the anteroposterior diameters' measurements from the present study and literature cited in the text: the external conjugate, the midlet, and outlet anteroposterior diameter increase. The obstetric conjugate decreases from the straight-leg to the squat position. The lumbar-sacral junction flexion increases the longitudinal hemi-diameter of the Michaelis area, and the sacral promontory flattens. The external conjugate becomes close to perpendicular to the lumbar spine anterior line, approaching the theoretical best birth position.

The sacral movement between the iliac bones is a composite question, not fully unfolded [21]. The thoracolumbar fascia layers and ligaments surround it, and it is assisted and regulated by the lumbosacral junction and sacroiliac joints [21,22]. Sacral base contra-nutation and nutation are the main obstetric labor movement reported during the dilation and expulsive labor stage. The sacral rotation between the iliac bones is permitted and limited by the balanced reciprocal tension of the long dorsal sacroiliac and sacrotuberous ligament, which are thoracolumbar fascia thickening [22]. The nutated sacrum (forward rotation) is more stable, anticipating joint loading, and occurs during load-bearing positions. The anteroposterior conjugate increases both in our study and in the simulation study squat position; in the MR studies, the obstetric conjugates decreased [2-4]. Hemmerich et al. had assumed the sacral base rotation movements were on the posterior lumbar-sacral zygapophyseal joints, not considering the sacroiliac but the hip joints [5]. They explicated the sacral promontory border rotates anteriorly, decreasing the internal conjugates, and the posterior sacrum moves posteriorly, increasing the Baudelocque diameter and flattening the lumbosacral angle (Figures 5A, 5B) [5,6].

The images obtained by MR allow the positioning of the digital calipers on the edge of the internal pelvis' bony surface. The external pelvimeter is joined with the tissues due to the spring's compressive pressure, resting firmly on the posterior bone surface with pelvic fascia and soft tissues' interposition, potentially affecting the measurement [14]. Biomechanical studies noted that the lumbosacral junction flexes, and the sacrum extends over the sacroiliac joints [6,22]. The posterior sacral border is the posterior reference of the external conjugate, as Baudelocque stated, not the tip of the spinous process of the fifth lumbar vertebra as it has been handed down [23]. The mechanisms mentioned may be involved and justify the increase in the pelvic inlet anteroposterior diameter measurement, detected in Hemmerich's and our study.

The internal and external conjugates were revealed to be well related [18]. The obstetric conjugate is one of the anteroposterior diameters of the pelvic inlet. It was selected as the obstructed labor best-predictor in MR and ultrasound research literature [10,18,24-26]. The external anteroposterior diameter of the pelvic inlet increases in the present study from the straight-leg to the kneeling squat position. It showed full static and dynamic correlation to the Michaelis area's transverse diameter, highlighted by a strong linear relationship. Correlations were found in both absolute and ROM values in different positions (Table 6), elucidating the Michaelis' transverse diameter's potential clinical role in obstructed labor screening, as previously reported [13,27].

**Figure 6:**
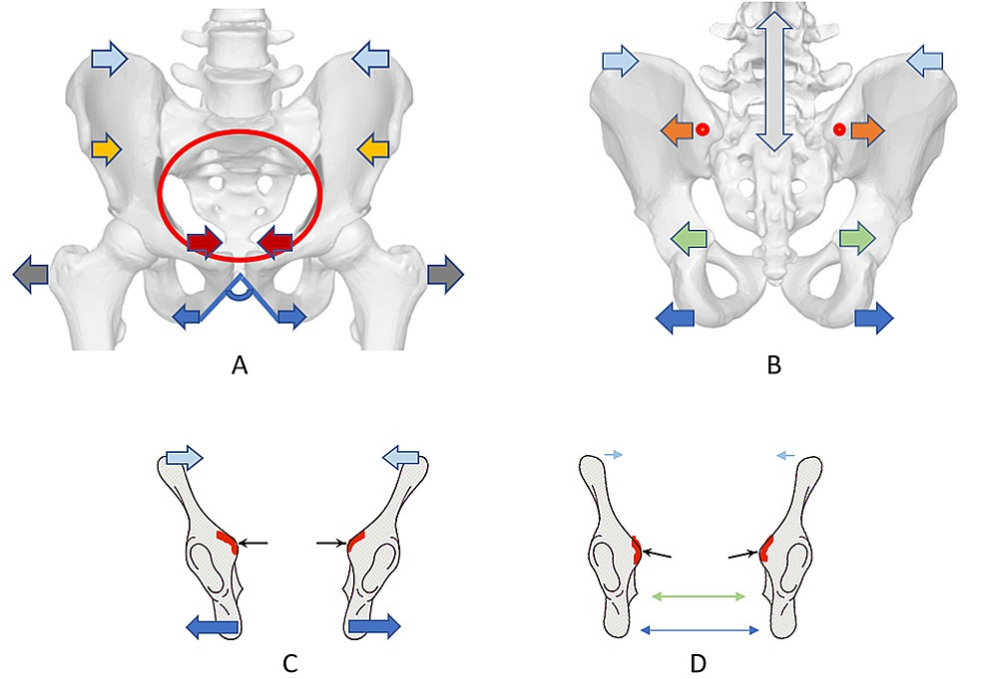
Pelvic diameters and inlet edge modification to the squat positions A, B: figures show the direction of pelvic diameters' measurement change to the squat position. A: anterior view of the pelvis. B: posterior view of the pelvis. Blue arrows: subpubic arch angle and inter-tuberosities diameter. Dark grey arrows: bi-trochanter diameter. Light blue arrows: iliac bi-crestal diameter. Green arrows: ischial bi-spinous diameter. Yellow arrows: iliac bi-spinous diameter. Dark red arrows: the base of Trillat's triangle. Grey arrows: longitudinal hemi-diameter of Michaelis sacral rhombus. Orange arrows: transverse diameter of Michaelis sacral rhombus. Red dots: the posterior superior iliac spines. Red line: pelvic inlet edge. C, D: hipbones orientation in the frontal plane. C: frontal section of the ischium-iliac bones in starting position. D: frontal section of the ischium-iliac bones in the squat position. Red line: pelvic inlet. Black arrows: arcuate lines' edge inclination. Blue arrows: inter-tuberosities diameter modification direction to the squat position. Light blue arrows: bi-crestal diameter modification direction to the squat position. Arrowed green line: midpelvic transverse measure in starting position. Arrowed blue line: pelvic outlet transverse measure in starting position.

All posterior diameters increased the measurement from standing to "all-4" position in our study. The posterior diameters increased further in the squat position, except the iliac bi-crestal distance decreased significantly (Figure 6A, 6B). The pelvic spaces enlarge in the hands-and-knees condition, confirming women's preference for this pain-relieving postural choice during the labor first stage [5,10]. Then, in the squat position, as the pelvis tilts posteriorly, the lower diameters (inter-tuberosities and bi-trochanteric diameter) further increased, and the lumbosacral junction flexes [6]. The longitudinal hemi-diameter of the Michaelis area measurements in shifting positions is comparable to a modified lumbar-sacral Schober's test, which evaluates the lumbar spine flexion ROM [19]. The Michaelis longitudinal hemi-diameter (flexion's degree of the sacral promontory) is closely related to the pelvic outlet's inter-tuberosities space (Table 5). They progressively increase their distance from the erect to the "hands-and-knees" and the kneeling squat position. The data are homogeneous with other studies that had confirmed the McRoberts' maneuver's value in the case of shoulder dystocia [3,6]. The legs' deep flexion on the pelvis opens up the lower pelvic diameters' space to the maximum and flattens the birth canal's curve as straight as possible (Figure 5B) [5,6].

According to Deseaeuve and colleagues' paper on 20 pregnant women, as the hip joints gradually flex, the pelvis tilts, and the lumbar spine flexes, increasing its vertical measure and the Michaelis longitudinal hemi-diameter [6]. From our results, the base of the sacrum widens the diameter between the posterior superior iliac spines (Michaelis' area transverse diameter): it appears as "the back opens" to give birth as described by traditional midwives [16,24]. The Michaelis sacral rhombus increases its size: it would correspond to a modification of the posterior pelvic inlet transverse diameter and a lumbosacral promontory flattening (Figures 5A, 5B, 6B). Simultaneously the iliac bi-crestal diameter decreases (mean 6.4 mm, SEM 1.01), and the inter-tuberosities space of the pelvic outlet increases (mean 14.9 mm, SEM 0.91), suggesting a downward inclination of the edge of the inlet: the iliac arcuate lines would become less prominent, as the sacral promontory (Figures 6A, 6B) [3,6]. The study results suggest that the change in the pelvic diameters' distances would lead to a change in the pelvic inlet posterior width and edge's angulation, facilitating the fetal presenting part's descent and protecting the maternal lateral pelvis' neurovascular structures from compression (Figures 6C, 6D).

The external diameters corresponding to the pelvic inlet, midlet, and outlet result in a direct linear correspondence in the discrete measurements obtained in the positions (Table 5). The change in one diameter shows a linear relationship to the other diameters of the birth canal's pelvic space, suggesting a coordinated movement of the pelvis as a whole. An MR pelvimetry study in supine static position confirmed a similar correlation between the obstetric conjugate, the sub-pubic arch angle, and the inter-spinous diameter [26].

The anterior pelvic diameters decrease in shifting positions, while the posterior diameters increase, showing an inverse direction of motion (Figure 6A, 6B). The width of the base of the Trillat's triangle corresponds to the pelvic midlet transverse diameter from the traditional pelvimetry [12]. The pelvic midlet space is the distance between the ischial spines, which lie in the posterior pelvis and show a mobility pattern like the outlet transverse diameter [3]. The Trillat's base negatively correlates with the inter-tuberosities diameter (Table 5), and its ROM relates with the external conjugate's ROM (Table 6). They refer to the anatomical function of the pubic symphysis, which is the fulcrum point of the birth canal's curve, around which the extension of the fetal head occurs, linked to the change of direction imposed by the second labor phase [28].

The DEP test cannot measure the anterior diameters in the squat position: probably they further narrow, according to correlations with other diameters and the direct anatomical connection between them and the bi-crestal diameter (Table 5). The inter-tuberosities diameter is anatomically part of the subpubic arch angle through the ischio-pubic branches; it increases in the squat position and the McRoberts' maneuver [5]. Consequently, we can assume that the superior border of the pubis and the anterior superior iliac spines, through the inguinal ligaments, decrease their distance with the legs' deep flexion on the pelvis, as occurs for the iliac crests (Table 2). The pelvic outlet transverse diameter is related to the pubic inferior angle and progressively increases to the squat position. The pelvic midlet ischial spines' diameter is correlated to the subpubic arch angle; they enlarge from the straight-leg to the kneeling squat position, as previous studies stated (Figures 5B, 6D) [2-5,26].

The iliac bi-crestal and the bi-trochanters diameters are well correlated in shifting positions. They demonstrated to mediate the relationships between diameters related to the pelvic inlet, midlet, and outlet (Table 5). Furthermore, they are the waist and hips circumference diameters, which catch the metabolic syndrome screening and prognosis better than body mass index [29]. The circumference can change over time, but diameter remains unchanged: the iliac bi-crestal and bi-trochanters diameters can be referred to as "structural" pelvic diameters. The bi-crestal diameter poorly increased in the hands-and-knees position (28% of subjects showed a decreasing motion value). It then decreased consistently in the squat position. The bi-crestal diameter does not recognize the simple patterns of motion of other diameters [16]. Similarly, the external conjugate's mean increased between p1 and p2 but decreased in 43% of subjects: both diameters showed wide variability in the measures' change behavior in shifting positions. Different pelvic types probably have different articular surface shapes and consequent different motion patterns, as reported by previous studies on the Michaelis sacral rhombus measurements [15,16].

The iliac bi-crest distance decreased from the "all-4" to the squat position correlating its motion significantly with the bi-trochanteric and the Michaelis rhombus' transverse diameter’s ROM, which coupled with the external conjugate's ROM (Table 6). Consequently, the pelvis space movement anatomical fulcrum appears to be placed between the sacral promontory and the iliac crest plane. The lower lumbar vertebrae would be relatively less mobile concerning the sacrum's movement. It is hypothesized that the iliolumbar ligaments, which are thickenings of the thoracolumbar fascia, could be a candidate to define the anatomical fulcrum, the relatively "fixed point" which regulates the dynamic behavior of the pelvic diameters in shifting positions [5,22]. More research is needed to elucidate the pivotal biomechanics movements during the parturition process entirely.

The study admits some limitations. The study group is large enough to draw general statistical conclusions, but each diameter has different movement patterns, some homogeneous and others non-homogeneous. The joint anatomy of the various pelvic types can justify such variability in pelvic mobility [5]. Previous studies showed the different shape and movement patterns of the Michaelis sacral area, explaining the differences in measurement between literature studies [15,16]. Argumentations about internal pelvic space modification in shifting positions are inferences based on indirect external measurements, albeit supported by a general agreement with biomechanical studies [2-8,20,26]. Our study was performed in a single-center. More research is needed, and multi-center studies could evaluate the dynamic external pelvimetry test on a larger number of patients and a more comprehensive range of clinical situations and pelvic type morphology. The relationship between external pelvimetry and obstetric outcomes is to be investigated.

## Conclusions

The study shows that the external pelvic diameters modify their space in shifting positions. Maternal posture is related to the birth canal's internal pelvic diameters allowing more room in the pelvis for safe childbirth. The Dynamic External Pelvimetry test could quickly assess pelvic mobility, has excellent reproducibility and reliability, and potentially is a screening test for the "contracted pelvis" and mechanical dystocia. The test is suitable for the daily obstetric clinical practice in any socio-economic and health resource environment. Further studies are needed to relate pelvic mobility to obstetric outcomes and to delve deeper into the physiology of pelvic biomechanics and biodynamics during pregnancy and childbirth.
